# Supplementary figures and images for: Mapping the ultrastructural topology of the corynebacterial cell surface
Source: PLoS Biol. 2025 Apr 15;23(4):e3003130. doi: 10.1371/journal.pbio.3003130 (PMC12021427; doi:10.1371/journal.pbio.3003130)

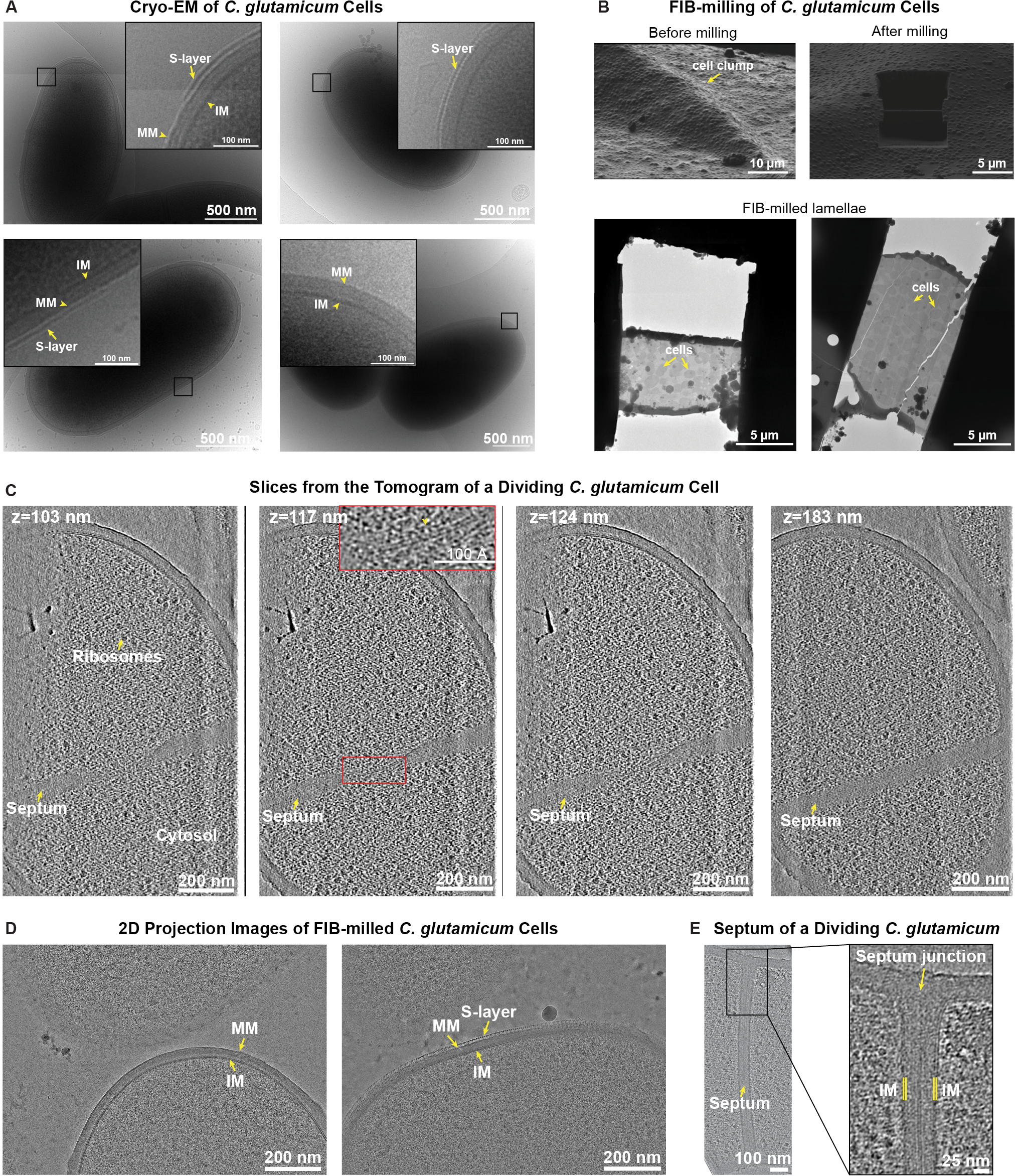

Supplement: S1 Fig — (A) Cryo-EM images of C. glutamicum cells deposited on grids without FIB milling. The S-layer decorates the C. glutamicum cell envelope in a patchy manner. S-layer (surface layer), MM (mycomembrane), and IM (inner membrane) are marked. (B) FIB-milling of C. glutamicum cells. Grids made for FIB-milling contained clumps of C. glutamicum cells, providing several suitable areas for milling. After milling, lamellae with a 150–200 nm thickness were retained for cryo-ET investigations. Each lamella contained multiple cells suitable for imaging. Although vitreous ice was observed in most lamellae, the edges of some lamellae showed signs of crystalline ice formation. (C) Slices from a tomogram of a dividing C. glutamicum cell (S1 Movie). The slices were bandpass-filtered to enhance contrast. The first slice was used as a reference point to calculate Z-values (marked in nm). Red inset shows a zoom of the site of division. (D) 2D projection images of FIB-milled C. glutamicum cells. The 2D projection images show high-contrast details of the cell envelope. The S-layer, MM, and IM are marked. The images were Gaussian-filtered to enhance contrast. (E) Septum of a dividing C. glutamicum cell. Ten 0.85 nm thick slices of the tomogram were averaged and bandpass-filtered to boost contrast. Zoomed view of the septum is shown on the right. (TIF) [file pbio.3003130.s001.tif]

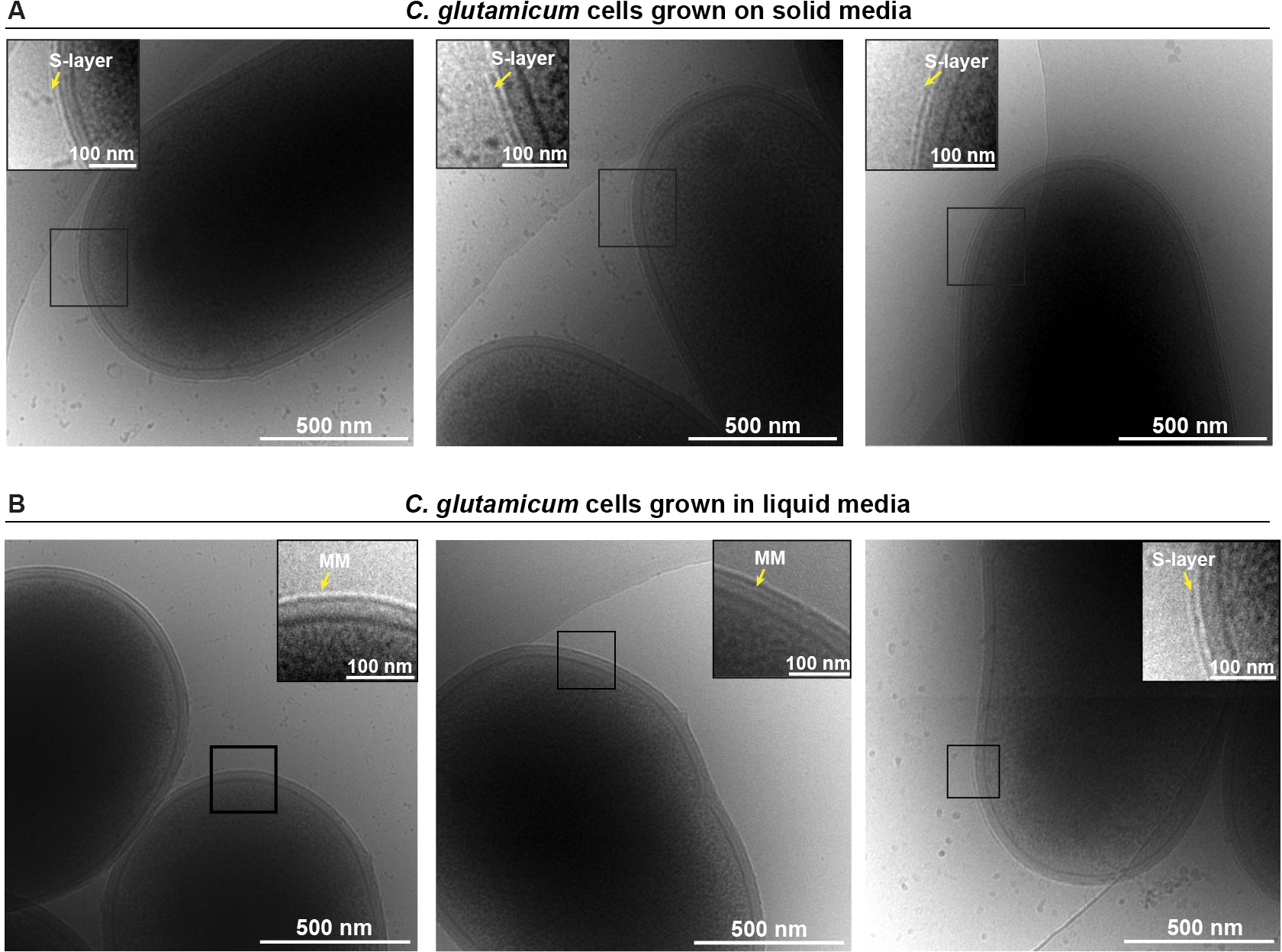

Supplement: S2 Fig — Cryo-EM images of C. glutamicum cells grown (A) on solid media, (B) in liquid media deposited on grids. S-layer coverage appeared to be increased in cells grown on solid media when compared to cells grown in liquid media, although the top and bottom parts of the cells are not interpretable in 2D images (and absent in milled lamellae). S-layer (surface layer) and MM (mycomembrane) are marked. (TIF) [file pbio.3003130.s002.tif]

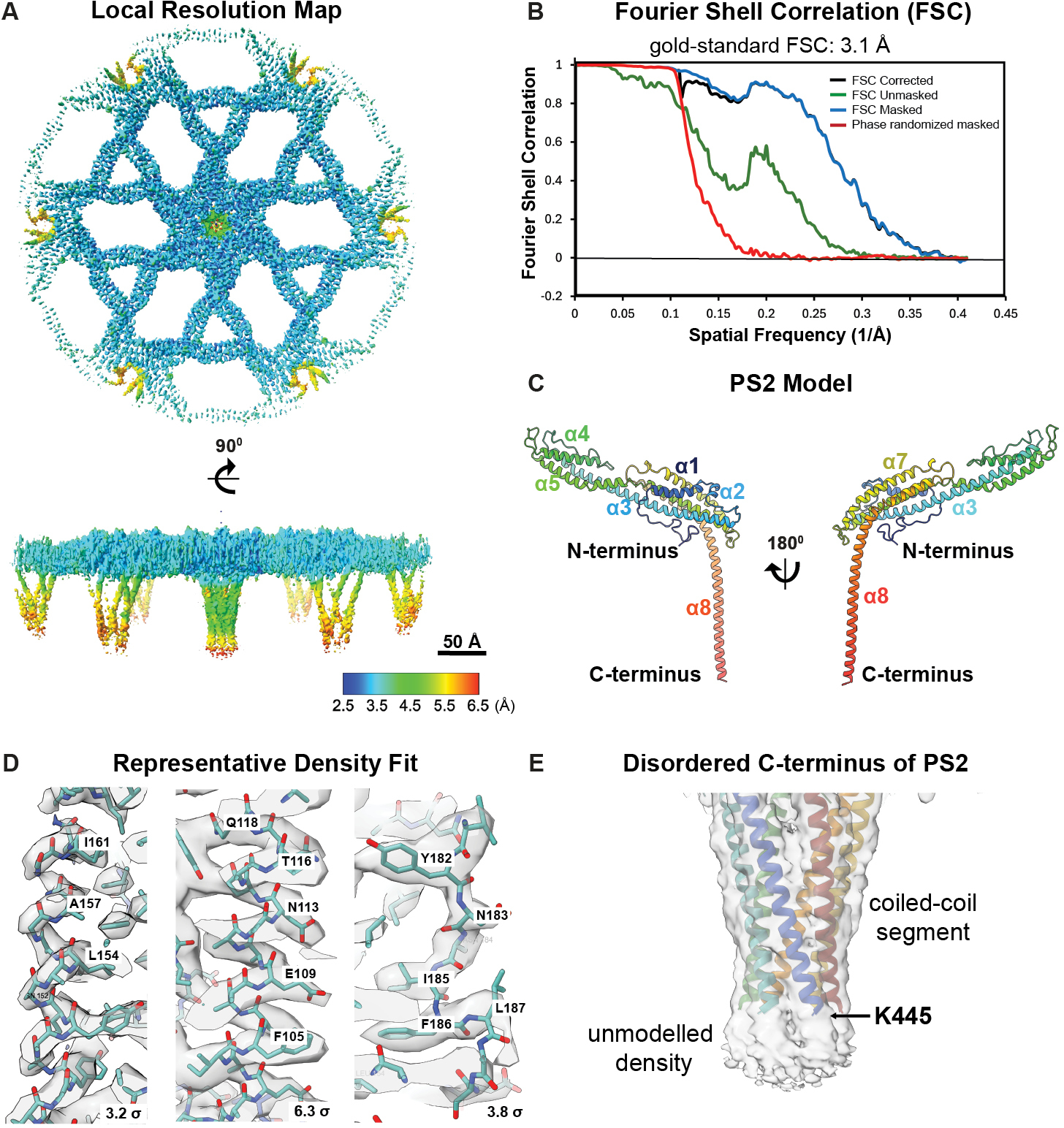

Supplement: S3 Fig — (A) Local resolution estimates plotted onto the cryo-EM map of the PS2 S-layer, colored based on the local resolution values shown in the color key (bottom right). (B) Fourier Shell Correlation (FSC) resolution estimation of the cryo-EM map. A gold-standard FSC estimates the global resolution of the map as 3.1 Å. The data underlying this plot can be found in S2 Data. (C) PS2 atomic model shown as a ribbon diagram in two orthogonal views, colored in a rainbow gradient with α-helices α1-α8 labeled. (D) Representative density fits of the PS2 model. (E) The density related to C-terminus of PS2 is disordered and could not be used for model building. The shown map has been Gaussian-filtered with a width of 0.9 Å to illustrate the unmodeled density at the tip of coiled-coil segment. (TIF) [file pbio.3003130.s003.tif]

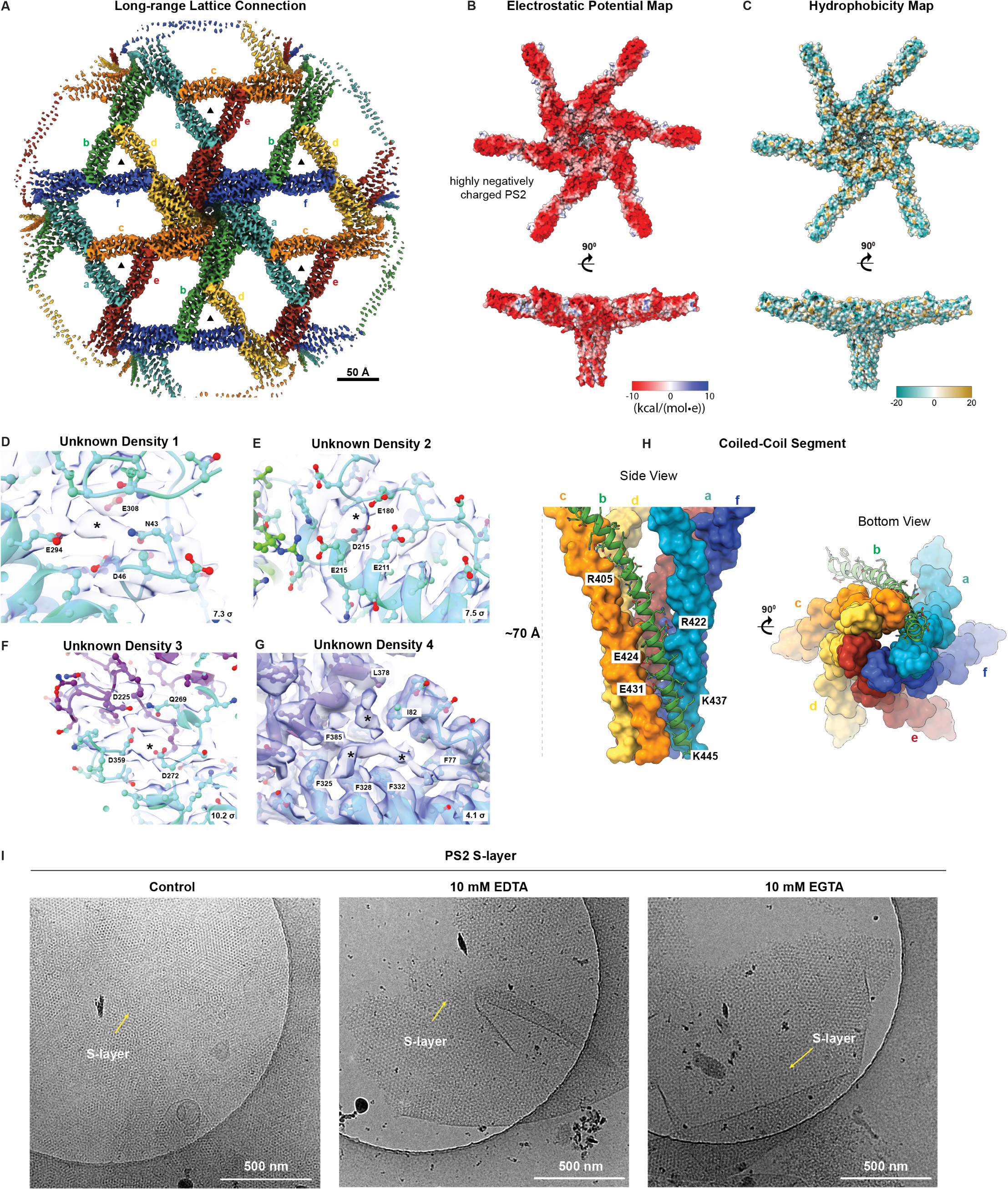

Supplement: S4 Fig — (A) The cryo-EM map of the PS2 S-layer is shown in color to illustrate the long-range lattice connections. Each color represents a different monomer of PS2, labeled from ‘a’ to ‘f’, repeated along the lattice. (B) Electrostatic potential map of the PS2 hexamer. (C) Hydrophobicity map of the PS2 hexamer. (D–F) Putative densities possibly corresponding to cations and (G) SDS detergent molecules are shown, with the respective sigma values of the maps shown in the bottom right. The potential densities are denoted with an “*”, and the surrounding residues are also labeled. (H) The coiled-coil segment (residues 405–445) is shown in side-view (left) and bottom-view (right). (I) Purified PS2 S-layer sheets incubated with EDTA (middle) and EGTA (right) show no discernible differences from native S-layers (left). (TIF) [file pbio.3003130.s004.tif]

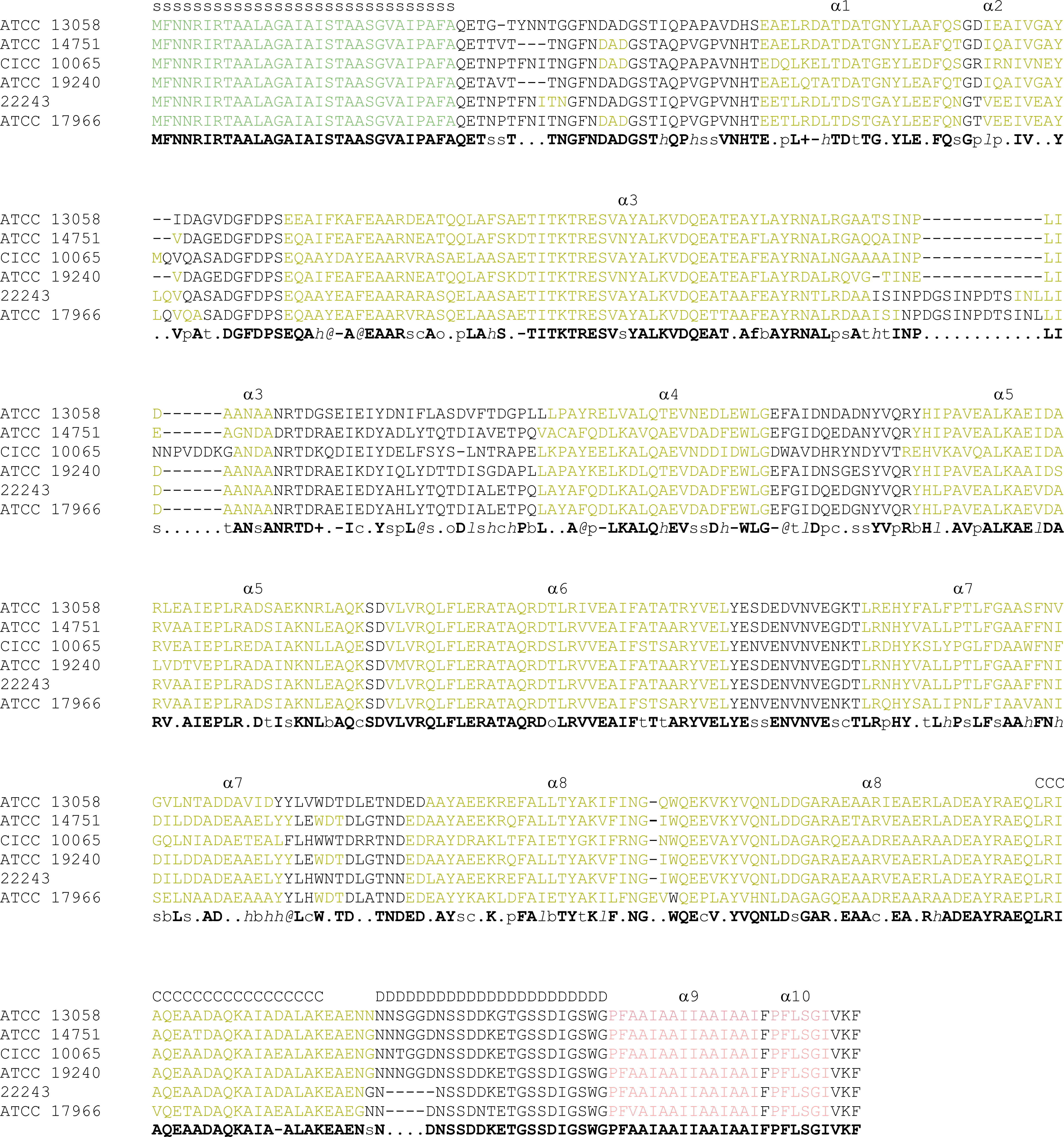

Supplement: S5 Fig — The alignment of PS2 sequences from the following strains is shown: ATCC 13058 (this study), ATCC 14751 (NCBI: AAS20296.1), CICC10065 (WP_040967778.1), ATCC 19240 (AAS20313.1), 22243 (AAS20307.1), and ATCC 17966 (AAS20302.1). Signal peptides in the sequences are highlighted in green, α-helices in yellow-green, and the MM-binding segment in light red. Coiled-coil (C) and intrinsically disordered (D) regions are indicated. Secondary structure was assigned based on our experimental model of C. glutamicum PS2 and AlphaFold2 models. The alignment was computed using PROMALS3D. In the consensus line, conserved amino acids are represented by bold and uppercase letters, with the following annotations: aliphatic (I, V, L): l; aromatic (Y, H, W, F): @; hydrophobic (W, F, Y, M, L, I, V, A, C, T, H): h; alcohol (S, T): o; polar residues (D, E, H, K, N, Q, R, S, T): p; tiny (A, G, C, S): t; small (A, G, C, S, V, N, D, T, P): s; bulky residues (E, F, I, K, L, M, Q, R, W, Y): b; positively charged (K, R, H): +; negatively charged (D, E): −; charged (D, E, K, R, H): c. (TIF) [file pbio.3003130.s005.tif]

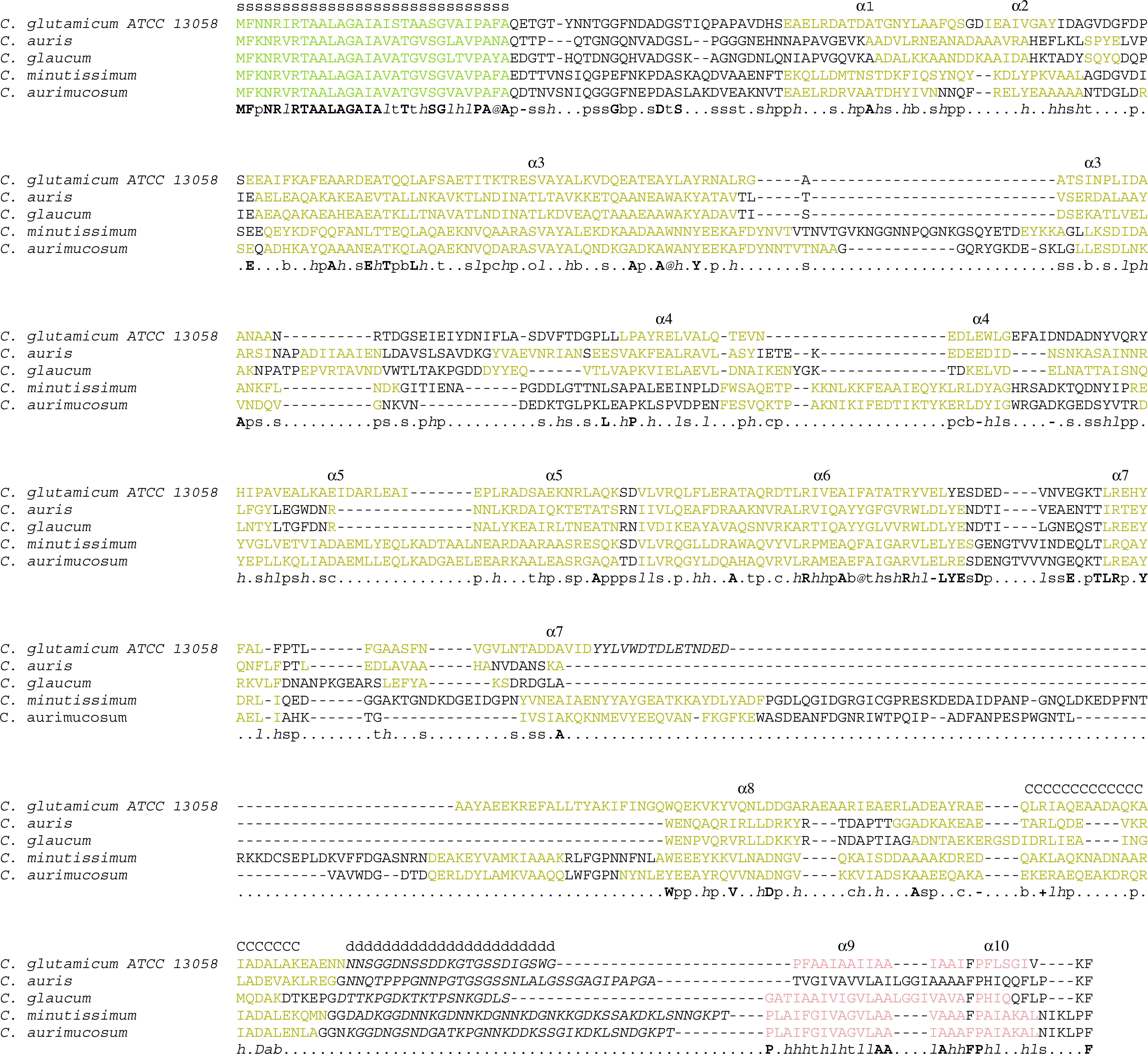

Supplement: S6 Fig — The alignment of PS2 sequences from the following species is shown: C. glutamicum ATCC 13058 (this study), C. auris DSM 44122 (WP_290341829.1), C. glaucum DSM 30827 (WP_095660674.1), C. minutissimum NCTC10289 (WP_115020885.1), and C. aurimucosum UMB1300 (WP_102234280.1). The sequences are annotated as in S5 Fig. (TIF) [file pbio.3003130.s006.tif]

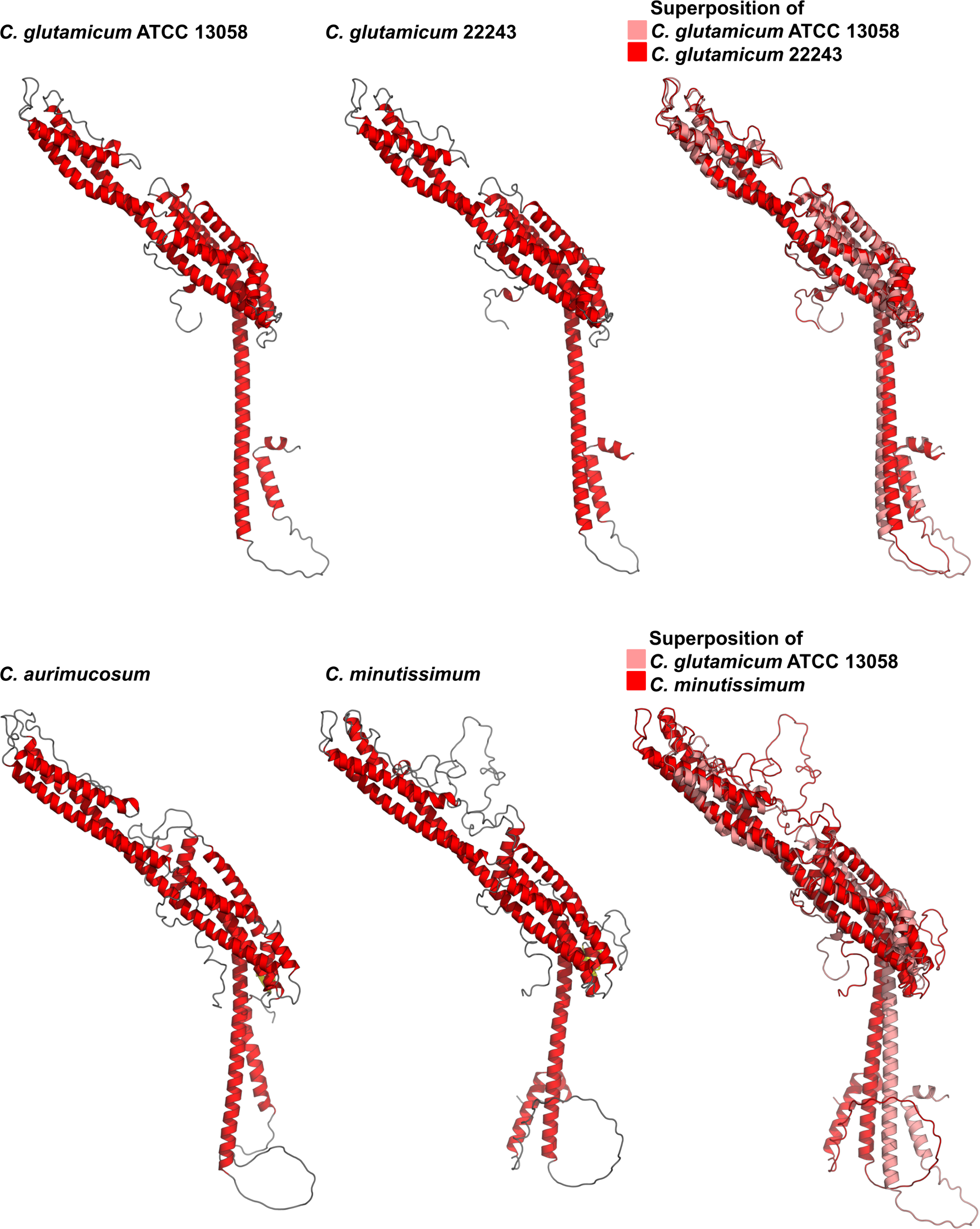

Supplement: S7 Fig — In all structures, α-helices are colored red. In the superimposed structures, one of the chains is shown in light red. (TIF) [file pbio.3003130.s007.tif]

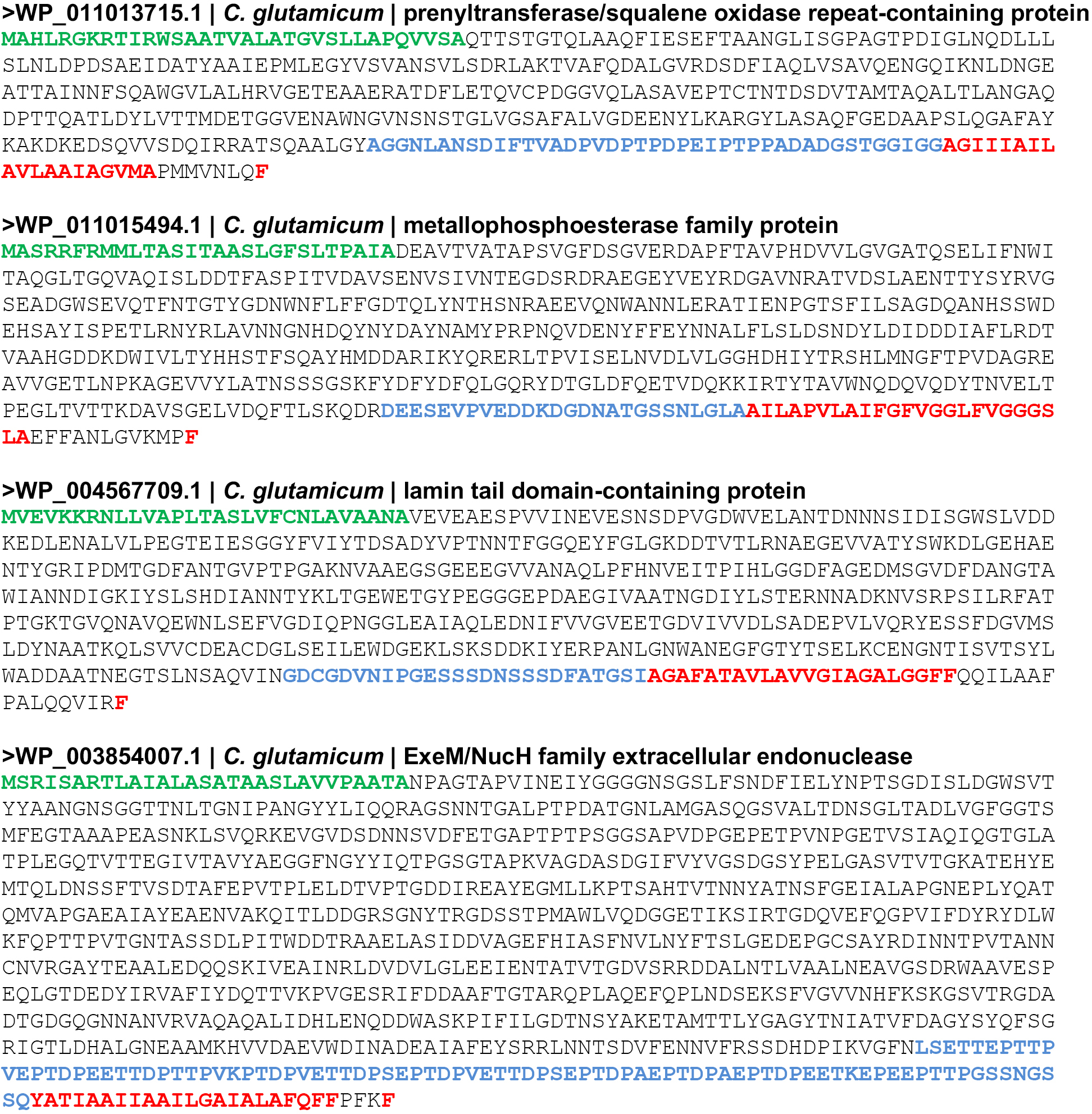

Supplement: S8 Fig — Predicted signal peptides are colored green, intrinsically disordered regions are colored blue, and the putative MM-binding segments are colored red. (TIF) [file pbio.3003130.s008.tif]
