# Supplementary material for: Mapping the ultrastructural topology of the corynebacterial cell surface
Source: PLoS Biol. 2025 Apr 15;23(4):e3003130. doi: 10.1371/journal.pbio.3003130 (PMC12021427; doi:10.1371/journal.pbio.3003130)
Supplement: S1 Table — Measurements were performed using the line profiles averaged over 20 nm along the cell envelope. Apart from the membranes, measurements were performed by measuring the peak-to-peak distances in the profiles. For reference, the peak-to-peak measurements for the membranes are as follows: MM without S-layer 3.1 ± 0.2 nm, MM with S-layer 3.0 ± 0.3 nm, IM without S-layer 3.8 ± 0.3 nm, and IM with S-layer 3.8 ± 0.5 nm. The membrane dimensions were measured directly from the micrographs. For each data point shown, six different measurements were performed, and standard deviations were calculated. The data underlying this table can be found in S1 Data. (PDF) [file pbio.3003130.s009.pdf]

S1 Table. Dimensions of cell envelope layers of *C. glutamicum*.

	average thickness (nm) ± standard deviation	
	**without S-layer**	**with S-layer**
**S-layer**	-	10.9 ± 0.9
**MM**	6.9 ± 0.3	7.1 ± 0.3
**OWZ**	6.3 ± 0.4	5.8 ± 0.6
**MWZ**	14.2 ± 1.4	13.4 ± 2.9
**IWZ**	10.5 ± 1.4	8.9 ± 1.5
**GL**	3.8 ± 0.5	3.8 ± 0.4
**IM**	7.2 ± 0.5	7.8 ± 0.6

Measurements were performed using the line profiles averaged over 20 nm along the cell envelope. Apart from the membranes, measurements were performed by measuring the peak-to-peak distances in the profiles. For reference the peak-to-peak measurements for the membranes are as follows: MM without S-layer 3.1±0.2 nm, MM with S-layer 3.0±0.3 nm, IM without S-layer 3.8±0.3 nm and IM with S-layer 3.8±0.5 nm. The membrane dimensions were measured directly from the micrographs. For each data point shown, 6 different measurements were performed, and standard deviations were calculated. The data underlying this table can be found in S1 Data.
