# Supplementary material for: Mapping the ultrastructural topology of the corynebacterial cell surface
Source: PLoS Biol. 2025 Apr 15;23(4):e3003130. doi: 10.1371/journal.pbio.3003130 (PMC12021427; doi:10.1371/journal.pbio.3003130)
Supplement: S2 Table — (PDF) [file pbio.3003130.s010.pdf]

S2 Table. Parameters for cryo-EM and cryo-ET data collection from FIB-milled cells.

Cryo-EM data collection statistics

Microscope	Titan Krios G3i
Detector	K3 (Gatan)
Energy Filter	BioQuantum energy filter
Data collection software	SerialEM
Magnification	42,000
Voltage (kV)	300
Slit Width (eV)	20
Defocus range (µm)	-3 to -5
Acquisition Mode	Counting super-resolution
Pixel size (Å)	1.064
Electron Exposure (e^{-}/$Å^2$)	66
Total images collected	56
Frames per movie	40

Cryo-ET data collection and image processing statistics

Microscope	Titan Krios G3i
Detector	K3 (Gatan)
Energy Filter	BioQuantum energy filter
Data collection software	SerialEM
Magnification	42,000
Voltage (kV)	300
Slit Width (eV)	20
Defocus range (µm)	-5 to -8
Acquisition Mode	Counting
Pixel size (Å)	2.13
Electron Exposure (e^{-}/$Å^2$)	122
Total number of tilts	61
Frames per tilt-movie	13
Tilt-series increment	±2°
Tilt-series scheme	dose-symmetrical (group size 6)

Tilt-series range	±60°
Tilt-series collected	15
Software motion correction	MotionCor2
Software tilt-series alignment	AreTomo
Software denoising	CryoCare
