# Supplementary material for: Mapping the ultrastructural topology of the corynebacterial cell surface
Source: PLoS Biol. 2025 Apr 15;23(4):e3003130. doi: 10.1371/journal.pbio.3003130 (PMC12021427; doi:10.1371/journal.pbio.3003130)
Supplement: S3 Table — (PDF) [file pbio.3003130.s011.pdf]

S3 Table. Cryo-EM data collection, image processing and model refinement statistics of the purified PS2 S-layer.

**Data Collection parameters of the purified PS2 S-layer**	
Microscope	Titan Krios G4
Detector	Falcon 4i (ThermoFisher Inc.)
Energy Filter	Selectrix X
Data collection software	EPU (ThermoFisher Inc.)
Magnification	105,000
Voltage (kV)	300
Slit Width (eV)	10
Defocus range (µm)	-1.6 to -2.2
Acquisition Mode	Counting super-resolution
Pixel size (Å)	0.611
Electron dose (e^{-}/Å^2)	50
Total movies collected	2,235
Frames per movie	40

**Data Processing statistics of the purified PS2 S-layer**	
Software used	CryoSPARC v4.5.3
Initial particle images	208,763
Final particle images	50,974
Pre-cropped box size (pixels)	720 x 720 x 720
Final box size (pixels)	360 x 360 x 360
Pixel size final reconstruction (Å)	1.222
Symmetry imposed	C6
Map resolution (Å)	3.1
FSC threshold	0.143
Map Sharpening B-factor (Å^2)	-56

Model Refinement of PS2 S-layer

Software	Phenix
Model resolution (Å)	3.6
FSC threshold	0.5
Model composition:	
Residue range	33-445
Domain Range	A-F
Protein residues	2478
B factors (min/max/mean) (Å^2)	
Protein	27.94/129.66/67.76
Rms deviations:	
Bond lengths (Å)	0.002 (0)
Bond angles (°)	0.478 (0)
MolProbity score	1.27
Clash score	4.48
Rotamer outliers (%)	0.62
Cβ outliers (%)	0.00
CABLAM outliers (%)	0.98
Ramachandran plot:	
Favoured (%)	97.81
Allowed (%)	2.19
Disallowed (%)	0.00
Rama-Z (Z-score, RSMD):	
whole (N= 2466)	1.49 (0.17)
helix (N= 1728)	2.17 (0.12)
sheet (N= 0)	--- (---)
loop (N= 738)	-1.87 (0.20)
