# Supplementary material for: Mapping the ultrastructural topology of the corynebacterial cell surface
Source: PLoS Biol. 2025 Apr 15;23(4):e3003130. doi: 10.1371/journal.pbio.3003130 (PMC12021427; doi:10.1371/journal.pbio.3003130)
Supplement: Attachment — Submitted filename: 150526_1_rebuttal_3231472_sp8drl.pdf [file pbio.3003130.s020.pdf]

Response to reviewers' comments for Isbilir *et al*

Black text – reviewer comment
Red text – author response
Blue text – manuscript excerpt

We thank the reviewers for their insightful comments and advice. In light of the reviewers' constructive suggestions, we have revised our manuscript as detailed below.

Reviewer #1 (Evidence, reproducibility and clarity (Required)):

Summary:
In this manuscript, the authors investigate the unique Mycobacteriaceae cell envelope using cryo-tomography/cryo-electron microscopy with Corynebacterium glutamicum as a model organism. Cryo-EM images of C. glutamicum cells successfully resolved previously observed densities corresponding to the MM, arabinogalactan, peptidoglycan, and inner membrane layers of the cell envelope along with the S-layer. The authors found that the S-layer is patchy in a manner dependent on growth phase (i.e. liquid versus solid growth). Intriguingly, when the S-layer was present, the leaflets of the MM appeared to be disrupted. The authors solved the structure of purified S-layer protein PS2 by cryo-EM, however they could not resolve the C-terminal membrane interaction domain. The authors found that PS2 is hexameric and different hexamers are linked by trimeric interface to create a porous structure. Phylogenetic analysis showed conservation of PS2 within corynebacteria and suggested a signature for MM-association.

Major comments:

(1) The S-layer structure is porous and the authors suggest that it may function as a molecular sieve or permeability barrier. This hypothesis should either be tested experimentally, or further discussion is needed regarding what small molecules (chemical features, size) would be able to penetrate.

This is a misunderstanding; we rather expect the opposite scenario in which the dimensions of the PS2 S-layer pores are too large to act as a molecular sieve. We are sorry for the confusion and have further clarified this part of the results and discussion.

Line 258: "The combination of hexameric and trimeric interfaces results in varying pores sizes of 6 Å, 27 Å, and 81 Å within the lattice (Fig. 3A). Some of these pores are relatively large and are reminiscent of the porous S-layer of *Deinococcus radiodurans*, which is also patchy on the cell surface (von Kügelgen et al., 2023). This suggests that *C. glutamicum* S-layer likely does not function as a molecular sieve, i.e. it has no protective role due to large pore dimensions and patchy cellular coating of the S-layer."

and

Line 470: "The large pores (especially the 27 Å- and 81 Å-pores) in the S-layer suggest that its role is not to protect the cells from invading molecules or phages."

(2) The authors show cryo-EM images of dividing C. glutamicum cells but don't make any statements as to the presence, morphology, and measurements of the different cell envelope layers. This analysis should be included.

We thank the reviewer for pointing this out. As suggested, we modified Figure S1 to highlight further details, and we have added the sentences below into the manuscript text.

Line 175: "To probe the plasticity of the cell envelope during the cell cycle, we analysed the cell envelope layers within the dividing septum (Fig. S1E). The thickness of the septum (~55 nm) was found to be greater than the usual thickness of the cell envelope (~42 nm on the same cell, see also Fig. 1A). The septum is composed of unseparated cell envelopes of the daughter cells that appear to contain a single 'outer' membrane, which is likely composed of mycolic acids. Presumably, this membrane will form the future MM once division is completed. Notably, the putative mycolic acid-containing bilayer within the septum was not connected to the MM on the other parts of the cell, whereas the remaining cell envelope layers appeared to be continuous with the rest of the cell. While IM and the putative future MM were clearly distinguishable, PG and AG could not be differentially identified in the dividing septum."

and

Line 422: "In addition to cell envelopes of non-dividing cells, the dividing *C. glutamicum* septum shows two daughter cell envelopes separated by a bilayer likely containing mycolic acids. Notably, this bilayer was not connected to the MM on the rest of the cell (Fig. S1E). This observation is in line with the previous studies showing that at septal junctions, a contiguous PG layer acts as a diffusion barrier for the MM, and during separation of daughter cells, the PG

in the septal junctions is displaced, allowing the bilayer at the septum to merge with the rest of the MM (Zhou et al., 2019)."

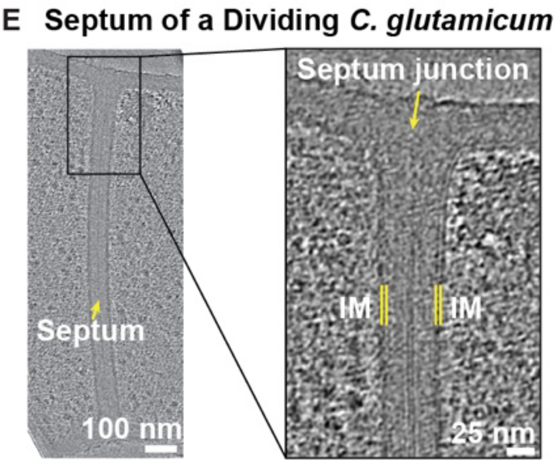

Figure S1. Cryo-FIB milling of *C. glutamicum* cells.
… **E)** Septum of a dividing *C. glutamicum* cell. Ten 0.85 nm thick-slices of the tomogram were averaged and bandpass-filtered to boost contrast. Zoomed view of the septum is shown on the right.

(3) The authors should include more discussion as to the patchiness or "wavy" MM near sites of PS2 contact. Cryo-EM of cells that express a variant of PS2 that lack the membrane anchoring domain would demonstrate that this is specific to PS2-membrane contacts. Minimally, providing some quantification for this phenotype would strengthen the claim (for instance, does the spacing between the perturbations match the expected scale of distance between S-layer membrane contacts).

We agree with reviewer that demonstrating the "wavy" nature of the MM requires further analysis. While it is our strong impression that the wavy nature is increased underneath the PS2 S-layer, we could not find a suitable metric to show this convincingly, i.e. all our analyses (real space averaging or averaging of power spectra) did not give clear-cut results. This is probably due to the inherent variability in the MM around the cell. In line with this, we have decided to tone down the relevant text in the manuscript.

Line 151: “Although we cannot be certain given the existing data, we suppose that this perturbation of the MM directly beneath the patchy S-layer could arise due to the interaction of the S-layer anchoring domain with the MM, which has been predicted to be present in the coiled coil part of the PS2 protein forming the S-layer using bioinformatics (Johnston et al., 2024).”

(4) The authors speculate on complete conservation of certain residues in the C-terminal domain of PS2 and hypothesize that they may be important for maturation or targeting of MM-associated proteins. Two additional examples of proteins with this motif are mentioned as evidence. Authors should search for this motif in pre-existing lists of MM proteins in the literature to test if this hypothesis is robust. Experiments to test if the conserved C-terminal residues of PS2 are required for export or assembly into an S-layer are feasible but optional given the scope of the paper.

We thank the reviewer for raising this point. Upon thoroughly re-examining the literature, we identified a previous study by Marchand et al. (J Bacteriol., 2012) that characterized MM-associated proteins in *C. glutamicum*. The proteins reported in this study as associated with the inner leaflet of the MM, including the mycoloyltransferases MytA and MytB, as well as those involved in pore formation, such as PorA and PorB, do not possess a phenylalanine as their terminal residue. This observation suggests that the invariant phenylalanine in PS2 does not represent a universal mechanism for targeting proteins to the MM. However, we also noted that several putative cell-surface proteins identified in this study, which feature a PS2-like C-terminal hydrophobic anchor preceded by a disordered segment, harbor a phenylalanine, proline, or lysine at their C-terminus. Additionally, the targeting of porins such as PorA, PorH, PorB, and PorC to the MM in *C. glutamicum* is known to depend on posttranslational O-mycoloylation. Based on these findings, we speculate that the conserved phenylalanine in PS2 may contribute to its anchoring and stabilization within the MM, rather than functioning as a universal targeting signal—a hypothesis we plan to investigate in future studies. We have revised the manuscript to incorporate these points and provide additional context.

Line 377: “To explore this hypothesis, we analysed MM-associated proteins of *C. glutamicum* identified in a previous study (Marchand et al., 2012). Proteins associated with the inner leaflet of the MM, such as the mycoloyltransferases MytA, MytB, MytC, MytD, and MytF, or those involved in pore formation, such as PorA and PorB, do not possess a phenylalanine as their

terminal residue, suggesting that the invariant phenylalanine in PS2 does not represent a general mechanism for targeting proteins to the MM. However, several putative cell-surface proteins with a PS2-like C-terminal hydrophobic anchor preceded by a disordered segment were found to harbor a phenylalanine, proline, or lysine at their C-terminus. Examples include a prenyltransferase/squalene oxidase repeat-containing protein (NCBI: WP_011013715.1) and a metallophosphoesterase family protein (WP_011015494.1) (Fig. S8). Based on this conservation, we identified additional putative MM-associated cell-surface proteins in *C. glutamicum* (Fig. S8), such as an ExeM/NucH family extracellular endonuclease (WP_003854007.1) and a lamin tail domain-containing protein (WP_004567709.1). Interestingly, the targeting of porins PorA, PorH, PorB, and PorC to the MM in *C. glutamicum* has been shown to depend on posttranslational O-mycoloylation, which facilitates their proper localization and integration into the mycomembrane (Carel et al., 2017). Whether O-mycoloylation is also involved in the targeting of PS2 remains an open question and warrants further investigation. We speculate that terminal residues such as phenylalanine, proline, and lysine may contribute to anchoring cell-surface proteins within the MM by stabilizing interactions with the hydrophobic membrane environment or acting as signals for specific sorting or assembly mechanisms."

(5) The authors do not draw the distinction between MM-associated and integral MM proteins (that contain a transmembrane domain). Is the C-terminal membrane anchoring domain of PS2 likely to span the entire bilayer or just be associated by a few amino acids?

The MM-anchoring hydrophobic segment is approximately 25 residues long across PS2 homologs, corresponding to a ~3.75 nm α-helix. In comparison, the MM has a thickness of 4–5 nm. This suggests that, while the MM-anchoring segment may not strictly qualify as a transmembrane domain integral to the MM, it is sufficiently long to embed deeply into the membrane and potentially span much of its bilayer thickness. To address this, we have added the following clarification to the manuscript:

Line 363: "The MM-binding segment is predicted by AlphaFold2 models to comprise an N-terminal hydrophobic α-helix and a short C-terminal amphipathic α-helix; however, in the MM, these may function as a single continuous helix. The MM-binding segment of PS2 homologs in Corynebacterium is consistently approximately 25 amino acid residues long,

corresponding to a ~3.75 nm α-helix—sufficiently long to nearly traverse the 4–5 nm thickness of the MM."

Minor comments:

(1) The authors comment that the thickness of the MM both with and without the S-layer is the similar and conclude that there is no change in mycolic acid length. The resolution of the technique is not sufficient to make this statement.

We agree with the reviewer in this point, while we can only measure the thickness of bilayer, we cannot comment on the thickness of each leaflet of the mycomembrane. Therefore, we have revised the text accordingly.

Line 144: "In 2D projection images of FIB-milled cells, the two leaflets of the MM were clearly resolved (Figs. 1C-D). The thickness of the MM in both cell envelopes with and without S-layer was between 4-5 nm (Table S1)."

(2) It would be helpful if the authors could comment if their membrane dimension measurements agree with previously published results in the main text of the manuscript. It is currently only included in the legend of Table S1.

Specifically regarding the MM, the measurements from both studies are quite similar; compare 4-5 nm from our study with 4.7 nm from Zuber *et al.*, 2008. As the reviewer suggested, we have revised the discussion to include the comparison of the measurements with Zuber *et al.*, 2008.

Line 413: "Our measurements are largely consistent with previous results (Zuber et al., 2008), except that in our data the IWZ was significantly thinner (~9.8 nm in this study vs. ~18 nm in Zuber et al., 2008), which is possibly due to strain differences. Moreover, our measurement of MWZ was slightly different because we could resolve OWZ as a separate layer, which was included into the MWZ measurement in the previous study (~15nm in this study vs. ~20.9 nm in Zuber et al., 2008) (Zuber et al., 2008)."

Reviewer #1 (Significance (Required)):

The manuscript provides compelling images and structures of the C. glutamicum cell envelope and S-layer protein PS2, respectively. These cryo-EM images of the cell envelope appear to agree nicely with pre-existing studies in the field. The introduction of the manuscript was well-written and the data in the manuscript is of broad interest to those who study the Mycobacteriaceae cell envelope. There is a lot of compelling data included in the paper, but the study would be strengthened by further analysis of the data as well as additional experiments to support some of the hypotheses suggested.

Thank you.

Reviewer expertise: bacterial genetics, bacterial cell envelope, protein transport

Reviewer #2 (Evidence, reproducibility and clarity (Required)):

Corynebacterium glutamicum is an organism with important industrial application, and it shares its complex cell-envelop architecture with organism of great relevance in human health such Corynebacterium diphtheriae and pathogenic mycobacteria. Using a cryo-EM and cryo-ET approaches together with phylogenetic studies, the authors provide of an in-deep structural characterization of the cell envelop of C. glutamicum. The authors map the different components of the cell envelope using high-resolution tomography, revealing unseen details of the outer wall zone, previously unsolved and attributed to the AG molecule. They provide with an atomic model of the PS2 S-layer at 3.1 A global resolution. The later discloses key features of the S-layer architecture, consisting of a hexagonal scaffold built by the PS2 protein, and its interaction with the mycolic membrane. The phylogenetic and bioinformatic studies show PS2 S-layer to be exclusively found within the Corynebacterium genus, although sporadically, and a correlation of PS2 presence/absence with other genetic differences. Despite PS2 homologues are shown to share common regions, which suggests all PS2 S-layers to exhibit a hexagonal lattice like the described in this study, but with divergent lattice parameters.

Major comments:

The authors provide with solid data supporting the structural models and conclusions stated. Text and figures are clear and nicely presented. I have however an important question regarding a the cryo-EM model. In Figure 3 B-C and Figure S3D-H, the authors depict protein details including hydrogen atoms, which make me question if the PS2 S-layer structure has been modeled including hydrogen atoms. The resolution of the cryo-EM data does not enable to model hydrogens that, if were included in the structure, should be removed of the coordinate file of the S-layer model and figures.

We agree with the reviewer that the current resolution of the cryo-EM map is not sufficient to model hydrogen atoms. The hydrogens were added to PS2 S-layer model during refinement in ISOLDE (Croll, 2018), and retained during Phenix real space refinement (Afonine et al., 2018; Liebschner et al., 2019). We agree with the reviewer that hydrogens should not be shown in the figures, since their positions have not been determined experimentally in our cryo-EM map. We have therefore removed these atoms from Figures 3 and S4.

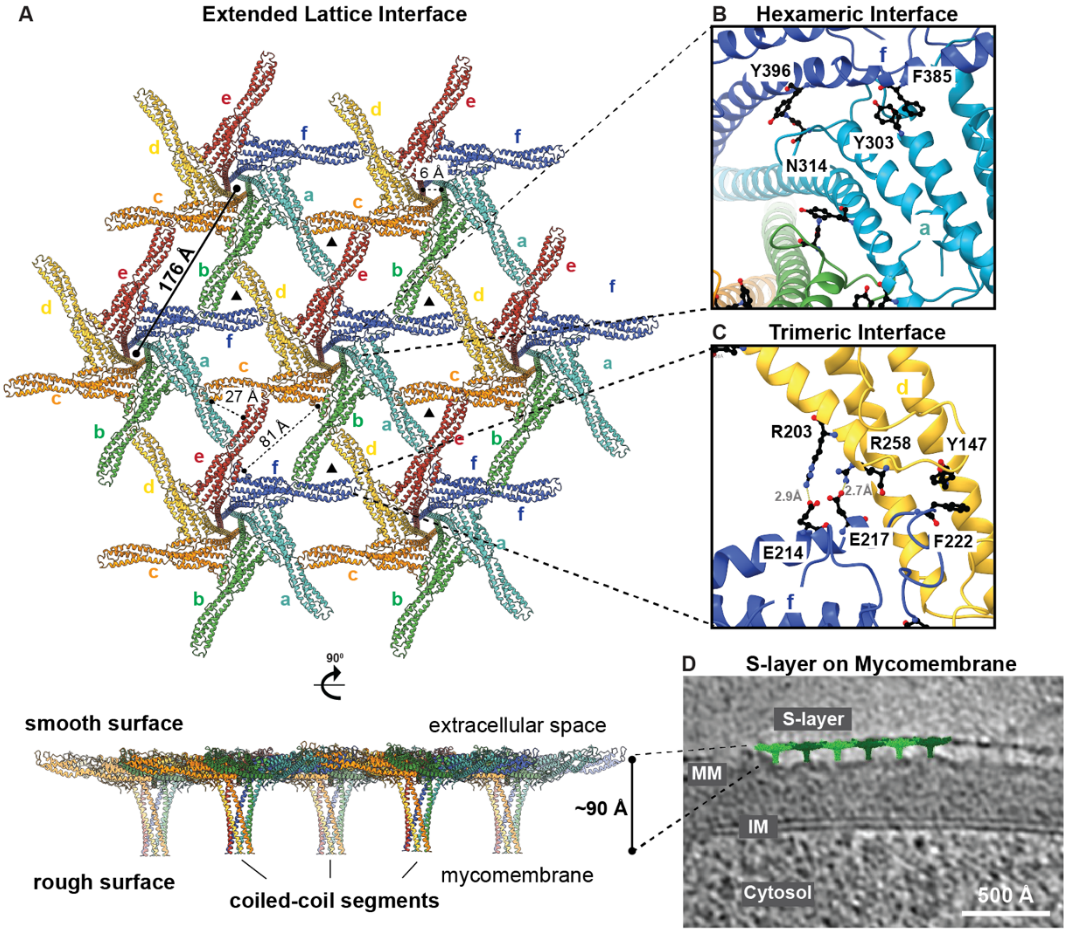

"Figure 3. The PS2 S-layer Lattice. …"

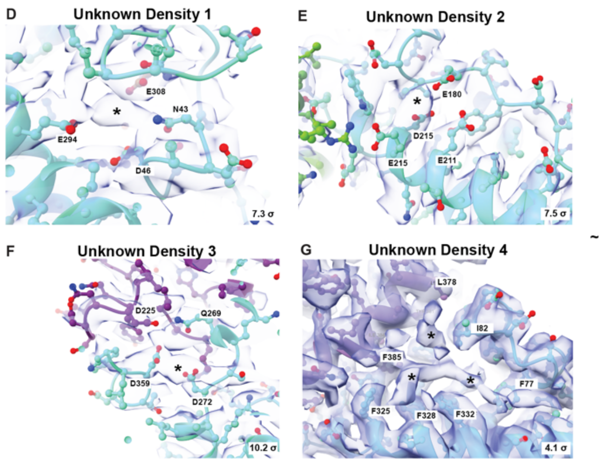

"Figure S4. Features of the PS2 S-layer lattice"

Minor comments

- Regarding the proposed calcium atoms at the S-Layer. The authors should provide further analysis to support the presence of calcium/divalent atoms proposed. Please show how is the coordination around the blobs spotted as potential calcium (or any other potential divalent that might be interacting at those positions). Does the coordination observed fit with the expected for a calcium/divalent binding site? Are the residues coordinating to those blobs well defined in density? Are the blobs of density of the potential cations observed across all the protomers of the PS2 S-layer? Figure 3D-F depicting the proposed cation-binding sites are too busy and unclear, they should focus on the proposed binding sites showing the interacting side/main-chains involved in the proposed coordination.

This is an interesting point. To investigate, we performed EDTA/EGTA treatment of the purified PS2 S-layer to see whether there would be any observable effect on the S-layer. We observed that S-layer lattices were still intact after EDTA or EGTA treatment. Therefore, we concluded that either cations do not play a role in stabilizing this S-layer or they are not accessible for chelation by EDTA or EGTA. This experiment unfortunately did not allow us to identify the ionic species. About the coordination: in the unknown densities 1 and 2 in the new Fig. S4, the coordination is clearer when compared to unknown density 3, however we cannot say for certain that these ions are calcium ions. Considering this, we have changed the text accordingly.

Line 237: "At the sequence level, the PS2 protein is enriched in acidic amino acid residues, giving it an overall negative charge, with an estimated isoelectric point of 4.25 (Fig. S4B-C). Consistent with this overall negative charge, we observed putative cationic densities at various locations along the PS2 sequence in the cryo-EM map, which are surrounded and stabilized by negatively charged amino acid residues (Figs. S4D-F). The identity of these cations cannot be ascertained at the current resolution of our cryo-EM map; however, previous studies on other bacterial S-layers suggest that they may correspond to calcium (Baranova et al., 2012; Herdman et al., 2022; Sogues et al., 2023). These cations may further stabilize the lattice, similar to other S-layers where cations were found to be essential for lattice formation (Baranova et al., 2012; Herdman et al., 2022; Sogues et al., 2023; von Kügelgen et al., 2021). To probe this further, we incubated purified PS2 S-layers with either 10 mM EDTA or 10 mM EGTA and examined the effect on the treated S-layers. Following the chemical treatment, S-layer lattices were still intact, with no observable differences under both conditions (Fig. S4I). This suggests that either

these putative cations do not play a major role in stabilizing the PS2 S-layer or they are not accessible for chelation by EDTA or EGTA under the chosen experimental conditions”

and

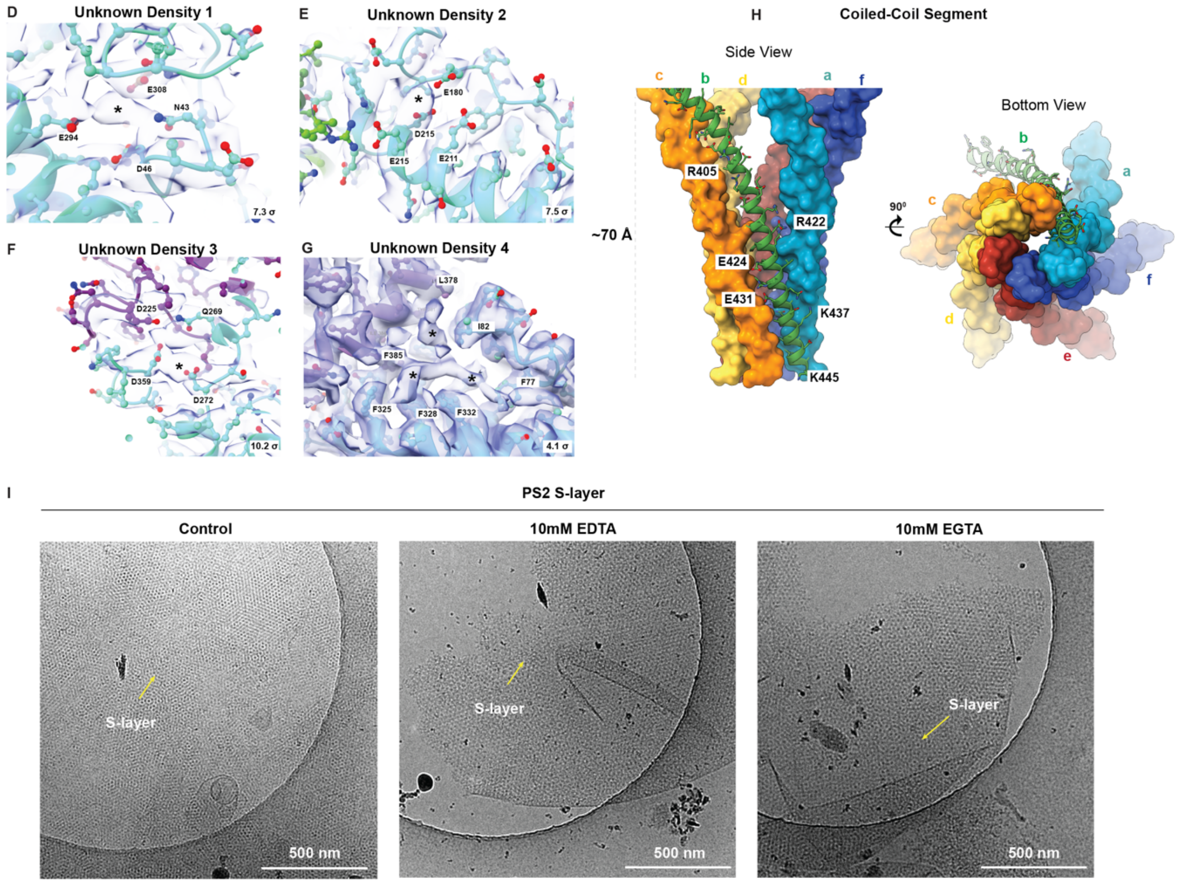

“Figure S4. Features of the PS2 S-layer lattice… D, E, F) Putative densities possibly corresponding to cations and **G)** SDS detergent molecules are shown, with the respective sigma values of the maps shown in the bottom right. The potential densities are denoted with an “*”, and the surrounding residues also labelled. **H)** The coiled-coil segment (residues 405-445) is shown in side view (left) and bottom view (right). **I)** Purified PS2 S-layer sheets incubated with EDTA (middle) and EGTA (right) show no discernible differences from native S-layers (left).”

- Regarding the potential SDS density. Looking at Figure 3G, it is not clear how the morphology of the density shown (with a T-shape) would fit a linear molecule of SDS (could be the view selected?). Have the authors performed any attempt of modelling the SDS molecule to assess this and/or those PS2 residues contributing to stabilize the SDS? Is this density consistently observed across the other interfaces of the hexamer? That would support their hypothesis.

This density is observed in the other interfaces of the hexamer as well, and it is also seen in maps that were produced from refinements without any symmetry applied, i.e. when the processing was performed in C1. Nevertheless, taking on board the criticism about the ambiguity of both the putative SDS and calcium densities, combined with the inconclusive results of our EDTA/EGTA treatment, we have changed the panel titles of Fig. S4D-G to "Unknown density 1-4" in revised the manuscript (see above), making sure to not claim more than what is revealed by the density.

Reviewer #2 (Significance (Required)):

As structural biologist I consider that this study constitutes an important advance in our understanding of the complex architecture and function of the cell-envelop of C. glutamicum. Knowledge that can help to better understand this intricate envelop present in other Mycobacteriaceae relatives, which include important human pathogen such as Mycobacterium tuberculosis or Corynebacterium diphtheriae. This study is most relevant for the scientific community investigating on the bacterial cell envelop (structure, evolution and function) as well as in host-pathogen interactions. Moreover, the cell envelop constitutes a target for bacteriostatics and thus, this study may be relevant for the scientific community working on antimicrobial development.

Thank you.

Reviewer #3 (Evidence, reproducibility and clarity (Required)):

Summary:

In the manuscript from Isbilir et al, the authors investigate the cell envelope of Corynebacterium glutamicum, a bacterium extensively used in biotechnological applications, using state-of-the-art cryo-electron microscopy methodologies as well as bioinformatics. They convincingly demonstrate that the C. glutamicum S-layer consists of hexagonal PS2 arrays and provide the underlying structural basis of this intriguing assembly. Bioinformatic analysis further revealed conserved and divergent elements of PS2 across Corynebacteria.

Major comments:

- My main point of criticism relates to the first part of the results, in which the authors attempt to characterize the cell envelope using cryo-electron tomography. From my own experience, plunge-freezing bacterial lawns often results in bad ice quality (crystalline ice) between the bacterial cells. This seems to be also the case here, looking at the 2D images in Fig. S1D revealing clear Bragg reflections. While often not a problem if interested in intracellular features, the authors are drawing conclusions on the cell envelope, which is in direct contact with these ice crystals, known to be destructive for ultrastructural features. For example, this could be the reason for the "wavy" mycomembrane in Fig. 1A and 1B as well as Fig. S1C. On top, it also might affect their observations of interrupted and discontinuous mycomembranes covered by an S-layer in Fig. 1D. The authors should discuss this limitation, and I would highly recommend rephrasing their conclusions made from this data more carefully.

We would like to thank the reviewer for their constructive criticism. We agree that it is difficult to vitrify a lawn of bacteria without formation of crystalline ice in all areas of the specimen. In our lamellae, we have primarily vitreous ice (see Fig. S1B, lower right panel for example) but the reviewer has correctly pointed out observed crystalline ice in some areas on the edges of the lamellae. As suggested, we included the following text in the legend to Fig. S1B to warn the readers about this potential shortcoming.

Line 562: “After milling, lamellae with a 150-200 nm thickness were retained for cryo-ET investigations. Each lamella contained multiple cells suitable for imaging. Although vitreous ice was observed in most lamellae, the edges of some lamellae showed signs of crystalline ice formation…”

The reviewer’s comment about the MM perturbations is well taken, this was also raised by reviewer 1. Although we attempted to quantify this effect by various image analysis tools, in the end we feel that it is not possible to make clear-cut conclusions about the MM-waviness based on our data. We have therefore toned down our interpretations about the “wavy” nature of the MM in the manuscript text (see also our response to reviewer 1 above).

Line 151: “Although we cannot be certain given the existing data, we suppose that this perturbation of the MM directly beneath the patchy S-layer could arise due to the interaction of the S-layer anchoring domain with the MM, which has been predicted to be present in the coiled coil part of the PS2 protein forming the S-layer using bioinformatics (Johnston et al., 2024).”

- The single-particle cryoEM data and the bioinformatic analysis are very well presented, analyzed in much detail, and convincing. While the authors state that the S-layer most probably does not serve to protect the cells from invading molecules or phages, additional experiments to figure out the function of the S-layer would be desirable. However, this might be beyond the scope of this paper but the authors should at least include a clearer discussion about potential function(s).

As suggested by the reviewer, we have extended the discussion about the potential function of the PS2 S-layer in *C. glutamicum*.

Line 465: “We also observed that S-layer coverage appeared to increase when *C. glutamicum* cells were grown on solid media (Fig. S2A-B). This suggests that the S-layer could be useful for the bacteria to grow in in a colony or in a surface-attached biofilm community, as shown for other bacteria including *Clostridium difficile* and *Tannerella forsythia* (Ðapa et al., 2013; Honma et al., 2007; Wong et al., 2023).”
and

Line 474: "…Slightly at odds with the large pores, it has been shown that the presence of the PS2 S-layer renders cells more resistant towards lysozyme (Sogues et al., 2024; Theresia et al., 2018). Although lysozyme is much smaller than the pore sizes, it is possible that the S-layer might biochemically sequester such undesirable molecules."

- The authors speculate about cations stabilizing the S-layer. To provide further evidence, an optional but straightforward experiment would be to treat the purified S-layer with EDTA and subsequently analyze it with negative stain EM or cryoEM.

As suggested, we incubated the purified PS2 S-layer with 10 mM EDTA or 10 mM EGTA and imaged the resulting specimens with cryoEM. We found intact S-layers in these treated samples, therefore, we have concluded that either cations do not play a role in stabilizing this S-layer or they are not accessible for chelation by EDTA or EGTA -

Line 246: "To probe this further, we incubated purified PS2 S-layers with either 10 mM EDTA or 10 mM EGTA and examined the effect on the treated S-layers. Following the chemical treatment, S-layer lattices were still intact, with no observable differences under both conditions (Fig. S4I). This suggests that either these putative cations do not play a major role in stabilizing the PS2 S-layer or they are not accessible for chelation by EDTA or EGTA under the chosen experimental conditions."

and

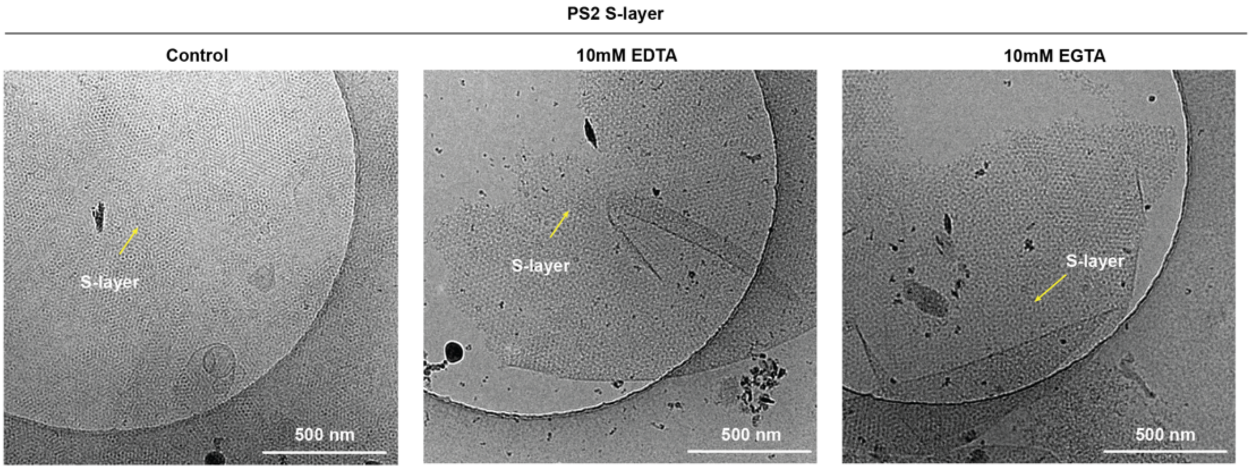

Figure S4. Cryo-EM of *C. glutamicum* cells. … I) Purified PS2 S-layer sheets incubated with EDTA (middle) and EGTA (right) show no discernible differences from native S-layers (left).

- The anchoring of the S-layer to the characteristic mycomembrane is only discussed very briefly. As this is a unique feature, it would be of high interest to understand how the anchoring is different from other S-layer carrying Gram-positive/negative bacteria.

We agree with the reviewer and have extended our discussion of this unique feature of the PS2 S-layer.

Line 359: "…the length of the coiled-coil stalk and the MM-binding segment is highly conserved among PS2 homologs across species (Figs S5-S6). This is in line with the fact that the underlying cell envelope architecture, including the MM, is preserved among different *Corynebacterium* species, necessitating the conservation of the MM anchoring segments in PS2. The MM-binding segment is predicted by AlphaFold2 models to comprise an N-terminal hydrophobic α-helix and a short C-terminal amphipathic α-helix; however, in the MM, these may function as a single continuous helix. The MM-binding segment of PS2 homologs in Corynebacterium is consistently approximately 25 amino acid residues long, corresponding to a ~3.75 nm α-helix—sufficiently long to nearly traverse the 4–5 nm thickness of the MM. Notably, this segment includes the last residue of PS2, a phenylalanine (F), which is remarkably conserved across all PS2 homologs (Figs S5-S6). While the functional significance of this invariant phenylalanine residue remains unclear, the conservation of the preceding residues, particularly the penultimate residue, which is typically either a proline (P) or lysine (K), suggests a potential functional role. It is plausible that these terminal residues collectively contribute to the sorting, export, and insertion of PS2 into the MM or help ensure its stable anchoring within the lipid-rich MM."

and

Line 444: "The PS2 S-layer protein has a distinctive mode of attachment to the prokaryotic cell envelope. In most archaea, S-layers are directly attached to the cytoplasmic membrane (Bharat et al., 2021), either through lipid modification of the SLP (von Kügelgen et al., 2021) or through the action of a secondary protein (von Kügelgen et al., 2024). In Gram-negative bacteria such as *C. crescentus*, S-layers are non-covalently attached to the O-antigen of lipopolysaccharide layer covering the outer membrane (von Kügelgen et al., 2020). In turn, in Gram-positive bacterial S-layers are non-covalently anchored via SLH domains to the PG-linked secondary cell wall polymers (Blackler et al., 2018). In other diderm bacteria that are positive for Gram-

staining such as *Deinococcus radiodurans*, the SLP HPI (Bharat et al., 2023) is lipidated at its N-terminus (von Kügelgen et al., 2023), allowing the protein to interact with the cell membrane. In the case of *C. glutamicum*, the attachment of the PS2 S-layer is achieved through the insertion of the C-terminal hydrophobic helix into the MM, which is a distinctive feature for bacterial S-layers that have been studied in detail using structural biology."

- Remove the word "accurately" in the second sentence of the second paragraph in the abstract.

Changed as requested.

Line 28: "Our cellular imaging allowed us to map the different components of the cell envelope onto the tomographic density."

- Remove the word "strong" in the last sentence of the abstract.

Done.

Line 41: "This study, therefore, provides an experimental framework for understanding cell envelopes that contain mycolic acids."

- As this is a back-to-back submission, the manuscript from Sogues et al. should be cited.

Done, as requested.

Line 191: "Purified S-layers were deposited on cryo-EM grids and vitrified using methods previously described for other S-layers (von Kügelgen et al., 2023, 2024), and specifically for the *C. glutamicum* S-layer concurrently with this study (Johnston et al., 2024; Sogues et al., 2024)."

and

Line 474: "…Slightly at odds with the large pores, it has been shown that the presence of the PS2 S-layer renders cells more resistant towards lysozyme (Sogues et al., 2024; Theresia et al.,

2018). Although lysozyme is much smaller than the pore sizes, it is possible that the S-layer might biochemically sequester such undesirable molecules."

Minor comments:

- Line numbers are missing, making the manuscript more complicated to review.

Sorry about that, the updated version of the manuscript has line numbers included.

- In the abstract, in the last paragraph of the introduction, and in the first sentence of the discussion, the authors use the term "high-resolution" in conjunction with their cryo-electron tomography imaging. This might be correct if you compare the data to light microscopy or conventional EM imaging. However, given the fact that the authors also used single-particle cryoEM, their cryoET data cannot be called "high-resolution," and they should remove this term as used here.

We agree with the reviewer and change the text accordingly:

Line 28: "Our cellular imaging allowed us to map the different components of the cell envelope onto the tomographic density."

and

Line 39: "Our structural and cellular data collectively provide a topography of the unusual *C. glutamicum* cell surface, features of which are shared by many pathogenic and microbiome-associated bacteria, as well as by several industrially significant bacterial species."

and

Line 102: "Building on these foundational studies, we have used *C. glutamicum* as a model for MM-containing organisms to perform characterisation of this unusual cell envelope."

and

Line 110: "By combining our S-layer structure with cryo-ET of the cell envelope and bioinformatics analyses, we provide further clues regarding the MM-anchoring mechanisms of the S-layer and offer insights into its conservation and evolution in corynebacteria."

and

Line 124: “To overcome this limitation, we employed FIB milling to create thin sections of the cells, which allowed us to obtain images with enhanced contrast of the cell envelope.”

and

Line 401: “In this study, we visualized the *C. glutamicum* cell envelope by imaging FIB-milled cells using...”

Reviewer #3 (Significance (Required)):

The single-particle cryoEM and bioinformatics analysis are convincing, but this manuscript resides at a rather descriptive level on the S-layer of C. glutamicum and some major comments should be addressed.

The findings in this manuscript are exciting for a specialized audience interested in bacterial cell surfaces/surface appendages and S-layers. On top, as C. glutamicum is widely used in biotechnological applications, the results have clear significance within this field.

Contrary to what the authors claimed, the general insights gained on cell envelopes containing mycolic acids are limited. Only very few insights reported here will advance our understanding of the cell envelope of important human pathogens such as Mycobacterium tuberculosis, as this manuscript focuses on the S-layer, which is absent from these strains.

Thank you for your comments, we have reworded the discussion section with more cautionary statements to present a balanced picture to readers of this manuscript.
