# Supplementary material for: Mapping the ultrastructural topology of the corynebacterial cell surface
Source: PLoS Biol. 2025 Apr 15;23(4):e3003130. doi: 10.1371/journal.pbio.3003130 (PMC12021427; doi:10.1371/journal.pbio.3003130)
Supplement: Attachment — Submitted filename: Isbilir_Response_Letter.pdf [file pbio.3003130.s022.pdf]

Response to reviewer's comments for Isbilir *et al,* "Mapping the ultrastructural topology of the corynebacterial cell surface" (PBIOLOGY-D-25-00518R1)

Black text – reviewer comment

Red text – author response

Blue text – manuscript excerpt

We thank the reviewer for their time in assessing our manuscript. In light of the reviewer's constructive suggestions, we have revised our manuscript as detailed below.

Reviewer #1:

In this manuscript, the authors study the S-layer of Corynebacterium glutamicum. They show that the S-layer is distributed patchily around cells, and that the MM layer is discontinuous in regions where there is an S-layer. They use Cryo-EM to make a structural description of S-layer oligomers. They use phylogenetic analysis to show that homologs of PS2, the S-layer protein, are only found in Corynbacterium genus, and are not found in most Coryne species.

The lack of conservation limits the significance of this work: S-layer is not found in other genus of the Mycobacteriaceae family. Is only found in some species of Corynebacterium. No data are presented that indicate a physiological role for the S-layer. In the discussion the authors mention that the S-layer contributes to lysozyme resistance - this is apparently the only information available that speaks to the significance of this structure - maybe this should be mentioned in the introduction?

In the abstract, the first paragraph implies that this work is relevant to mycobacterial pathogens. However, this work is focused on the S-layer, and the authors themselves show that the S-layer protein is not found in any Mycobacteria. The abstract and Introduction (eg, likes 94-97) should be re-written so as not to mis-lead readers about the impact of this work. This work does not provide insights into the cell envelope of mycobacteria. I suggest, in the Abstract and Introduction, focusing more on the general roles of S-layers, which should involve a literature review of other organisms that contain S-layers, not of the mycobacteria.

Taking on board this fair criticism, we have toned down detailed aspects related to mycobacteria, mentioning them only in one sentence in the introduction. We hope that this extensively modified abstract and introduction will accurately reflect the scope of the article.

In particular, we have removed all references to mycobacteria in the abstract -

Line 21: "*Corynebacterium glutamicum* is a diderm bacterium extensively used in the industrial-scale production of amino acids. Corynebacteria belong to the bacterial family *Mycobacteriaceae*, which is characterized by a highly unusual cell envelope with an outer membrane consisting of mycolic acids, called mycomembrane. The mycomembrane is further coated by a surface (S-)layer array in *C. glutamicum*, making this cell envelope highly distinctive. Despite the biotechnological significance of *C. glutamicum* and biomedical significance of mycomembrane-containing pathogens, ultrastructural and molecular details of its distinctive cell envelope remain poorly characterized."

Furthermore, we have extended our introduction to highlight S-layers, as suggested by the reviewer -

Line 67: "Many diderm and monoderm bacteria have a proteinaceous, paracrystalline surface (S)-layer as the outermost coat of their cell envelope (von Kügelgen et al., 2023; Lanzoni-Mangutchi et al., 2022; Sogues et al., 2023; Bharat et al., 2017; Fagan & Fairweather, 2014; Baranova et al., 2012; Gambelli et al., 2024; Fioravanti et al., 2019; Wang et al., 2023). S-layers are composed of repeating units of S-layer proteins (SLPs) that form a two-dimensional lattice encapsulating the cell (Johnston et al., 2024; Sleytr et al., 2014). Although maintaining an S-layer requires significant metabolic investment, its preservation throughout evolution in most bacteria and archaea suggests that it provides considerable selective advantages (Johnston et al., 2024). While some S-layers have not yet been associated with specific functions, many other S-layers play key roles in maintaining cell shape, regulating cellular defence, and promoting pathogenicity (Albers & Meyer, 2011; Bharat et al., 2021; Fagan & Fairweather, 2014)."

Line 26 - this is inaccurate, many ultrastructural details of the mycobacterial cell envelope are well described. Please be more specific about what is unknown and what you are addressing.

We agree with this point and therefore modified the text to mention only *C. glutamicum* specifically.

Line 24: "The mycomembrane is further coated by a surface (S-)layer array in *C. glutamicum*, making this cell envelope highly distinctive. Despite the biotechnological significance of *C. glutamicum* and biomedical significance of mycomembrane-containing pathogens, ultrastructural and molecular details of its distinctive cell envelope remain poorly characterized."

Introduction can be edited: background information at the introductory undergraduate level is not required.

Sorry for the simplistic language and have made the text briefer as suggested by the reviewer, limiting introduction of the cell surface to one sentence.

Line 62: "In monoderm bacteria, the inner membrane (IM) is covered by a thick peptidoglycan (PG) layer, while diderm bacteria have a thinner PG layer and are enveloped by a second lipid bilayer called the outer membrane (OM), which is often decorated by lipopolysaccharides (LPS) (Viljoen et al., 2020)."

Fig S1 - the insets in panel A are too small to see anything. Since the insets are where the important information is, maybe make them bigger and make the zoom-out pics smaller?

We have doubled the sizes of the insets in Figure S1A and also doubled the size of the inset in panel S1C (the inset in the 117 nm slice).

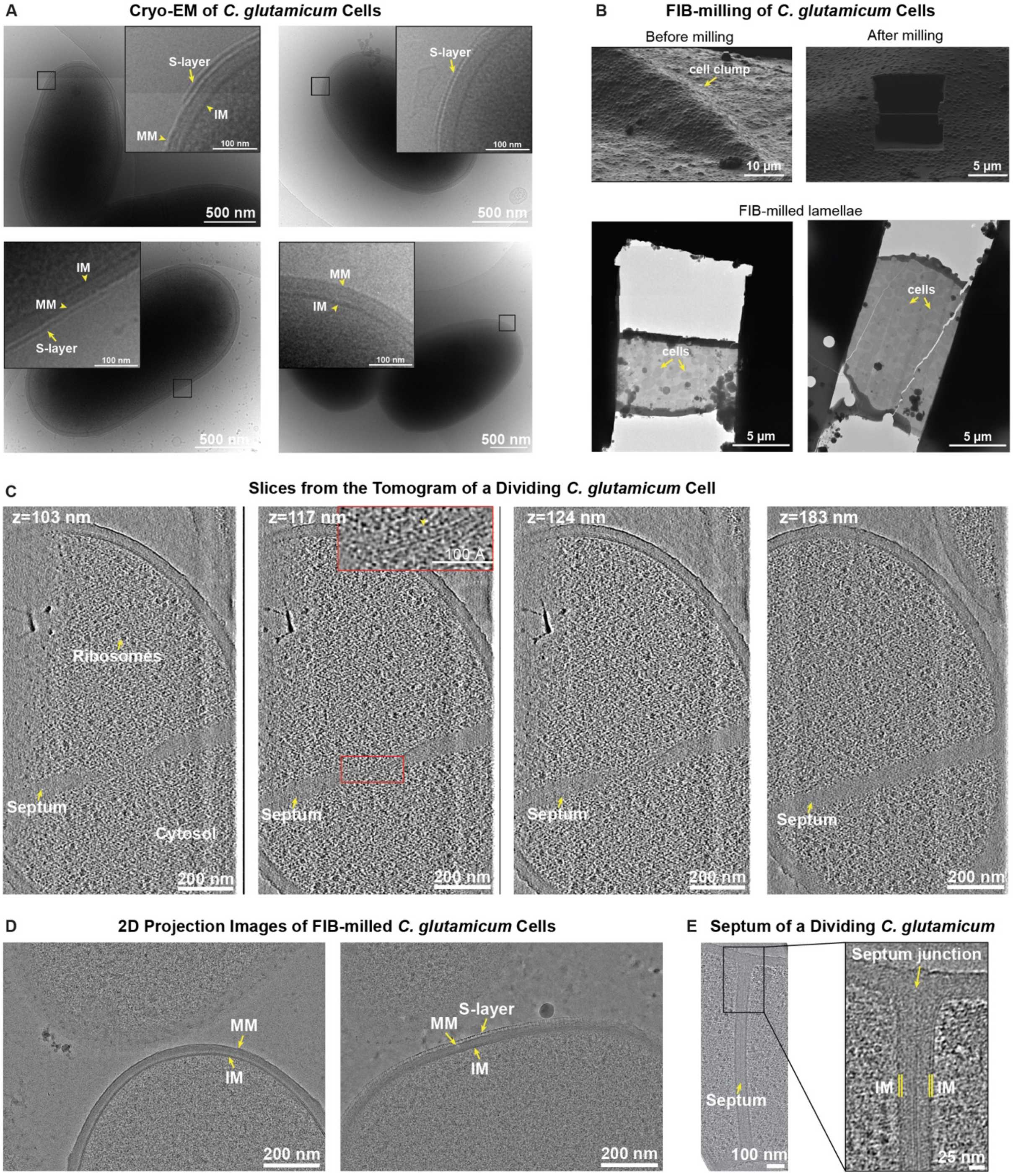

Figure S1. Cryo-FIB milling of *C. glutamicum* cells.

Fig 4 line 323 - please explain briefly how this phylogenetic tree was built in the results section - what models are used to infer relationships? What data are used?

As the reviewer requested, we have added a brief explanation of the phylogenetic tree generation to the results section.

Line 321: "To investigate the evolutionary distribution of PS2, we constructed a phylogenetic tree of 1,585 *Corynebacterium* species (Fig 4A) using the de novo workflow (de_novo_wf) of the Genome Taxonomy Database Toolkit (GTDB-Tk) (Chaumeil et al., 2022). This method infers evolutionary relationships based on concatenated multiple sequence alignments of 120 conserved bacterial marker genes, providing a robust phylogenetic framework. The resulting tree reveals a sparse yet widespread distribution of PS2, suggesting that the common ancestor of *Corynebacterium* likely possessed PS2 and, by extension, was coated with an S-layer assembled from it (Fig 4A)."

To be absolutely certain there are no confusion, we have included the tree files for the phylogenetic trees with this submission and cited their locations in the Figure legends-

For S4 Data. Fig4A.tree-
Line 547: "…The data underlying this tree can be found in S4 Data."

For S5 Data. Fig4B.tree-
Line 549: "The data underlying this tree can be found in S5 Data."

Please spell out what GTDB stands for in lines 538 and 846.

GTDB or Genome Taxonomy Database was only spelt out in the introduction. As reviewer suggested, we have spelt out GTDB in the rest of the text as well.

Line 79: "Corynebacteria are a part of the family *Mycobacteriaceae* (based on the Genome Taxonomy Database, GTDB (Parks et al., 2022))…"

Line 539: "**A)** The presence of PS2 in species across the *Corynebacterium* species is indicated by green dots on a GTDB (Genome Taxonomy Database, (Parks et al., 2022)) species-level tree."

Line 718: "…(*de_novo_wf*) of the GTDB-Tk 2.4.0 software toolkit (Genome Taxonomy Database) (Chaumeil et al., 2022; Parks et al., 2022)…"

Lines 324-325 - can you please explain the logic behind this conclusion more? Why is your assumption that the common ancestor contained an S-layer? Instead of that S-layer genes could be horizontally transferred?

Since PS2 is widely distributed among *Corynebacterium* species, we conclude that their common ancestor likely possessed the PS2 gene. If PS2 had been acquired through horizontal gene transfer (HGT), multiple independent uptake events would have been required across different species. While this scenario remains possible, it is less parsimonious than vertical inheritance from a common ancestor, making the latter the more likely explanation. Furthermore, our sequence similarity and structural searches did not identify PS2-like proteins outside *Corynebacterium*, further supporting the hypothesis that PS2 originated within this genus rather than being acquired through HGT. We have added a sentence to the manuscript to address this point.

Line 325: "The resulting tree reveals a sparse yet widespread distribution of PS2, suggesting that the common ancestor of *Corynebacterium* likely possessed PS2 and, by extension, was coated with an S-layer assembled from it (Fig. 4A). This aligns with our earlier findings that PS2 is restricted to *Corynebacterium* and likely emerged within this lineage."

Lines 486-490 - this conclusion is unsubstantiated. You are trying to make conclusions about the composition of the membrane from the sequence of proteins that insert into that membrane? This doesn't make sense to me - if there is some way to make such inferences, please provide citations. There are already direct data about the composition of mycomembranes in these species. Also, your paper is not about the mycomembrane, so this seems like a strange final statement.

Yes, this general point about the focus of our article is well-taken. Based on the reviewer's concern, we have removed the statement.

Line 493: "… further implies that while the S-layer has diverged within corynebacteria, the MM-anchoring mechanism has likely remained the same."
